# Metabolic and Nutritional Issues Associated with Spinal Muscular Atrophy

**DOI:** 10.3390/nu12123842

**Published:** 2020-12-16

**Authors:** Yang-Jean Li, Tai-Heng Chen, Yan-Zhang Wu, Yung-Hao Tseng

**Affiliations:** 1 Department of Pediatrics, Kaohsiung Municipal United Hospital, Kaohsiung 80455, Taiwan; ptstyle1986@gmail.com; 2 Department of Pediatrics, Division of Pediatric Emergency, Kaohsiung Medical University Hospital, Kaohsiung Medical University, Kaohsiung 80708, Taiwan; ijw168@yahoo.com.tw (Y.-Z.W.); smapten@gmail.com (Y.-H.T.); 3 School of Post-Baccalaureate Medicine, College of Medicine, Kaohsiung Medical University, Kaohsiung 80708, Taiwan

**Keywords:** spinal muscular atrophy, metabolomics, nutrition, therapeutics, biomarkers

## Abstract

Spinal muscular atrophy (SMA), the main genetic cause of infant death, is a neurodegenerative disease characterized by the selective loss of motor neurons in the anterior horn of the spinal cord, accompanied by muscle wasting. Pathomechanically, SMA is caused by low levels of the survival motor neuron protein (SMN) resulting from the loss of the *SMN1* gene. However, emerging research extends the pathogenic effect of SMN deficiency beyond motor neurons. A variety of metabolic abnormalities, especially altered fatty acid metabolism and impaired glucose tolerance, has been described in isolated cases of SMA; therefore, the impact of SMN deficiency in metabolic abnormalities has been speculated. Although the life expectancy of these patients has increased due to novel disease-modifying therapies and standardization of care, understanding of the involvement of metabolism and nutrition in SMA is still limited. Optimal nutrition support and metabolic monitoring are essential for patients with SMA, and a comprehensive nutritional assessment can guide personalized nutritional therapy for this vulnerable population. It has recently been suggested that metabolomics studies before and after the onset of SMA in patients can provide valuable information about the direct or indirect effects of SMN deficiency on metabolic abnormalities. Furthermore, identifying and quantifying the specific metabolites in SMA patients may serve as an authentic biomarker or therapeutic target for SMA. Here, we review the main epidemiological and mechanistic findings that link metabolic changes to SMA and further discuss the principles of metabolomics as a novel approach to seek biomarkers and therapeutic insights in SMA.

## 1. Introduction

Spinal muscular atrophy (SMA) is a congenital neuromuscular disease characterized by progressive muscle weakness resulting from the degeneration of motor neurons (MN) in the spinal cord [1]. Although SMA is considered a rare disease and the global incidence of live births is estimated to be about 1/10,000, SMA is still the second most common autosomal recessive genetic disease and the most common monogenic disorder that causes early infant death [2]. The carrier frequency varies from 1 in 38 to 1 in 72 among different ethnic groups, with a pan-ethnic average of 1 in 54 [3,4].

In a pathological view, SMA is resulted from an insufficient level of a 38 kDa protein, called the survival motor neuron (SMN), as a result of homologous deletion or mutation of the *Survival of Motor Neuron 1* (*SMN1*) gene [5]. Subsequent studies showed that two genes encode SMN protein in humans: *SMN1* and a 99% identical copy in sequence, known as *SMN2*. Indeed, *SMN2* mainly differs from *SMN1* by a single nucleotide (C-to-T) substitution in the exon 7 [6]. Such a critical variant results in exon 7 exclusion in most transcripts (90%) of *SMN2*, SMN∆7. Unlike the *SMN1* gene, *SMN2* can only produce 10 % full-length (FL) SMN [7]. Given that the residual FL-*SMN2* transcripts can compensate for defect *SMN1* to a limited extent, the SMA severity is partially rescued by *SMN2* copy numbers [8]. However, the correlation between this phenotype and genotype is not absolute, and recent studies have pointed out that other potential cellular mechanisms may also be involved in modifying the clinical severity of SMA [9].

**Figure 1:**
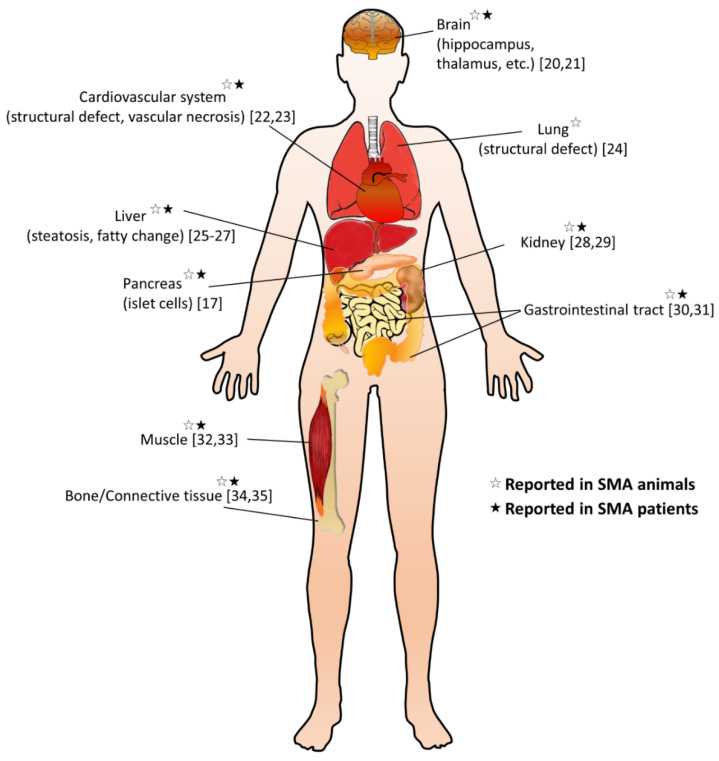
Overview of non-neuromuscular systemic pathology in spinal muscular atrophy (SMA). A summary of multi-organ involvement has been reported in SMA animal models and/or patients [17,20,21,22,23,24,25,26,27,28,29,30,31,32,33,34,35].

It is still unclear whether the pathogenesis of SMA is caused by a specific pattern or a combination of dysregulated effects. The cell-autonomous effects due to SMN deficiency are the main causes of MN degeneration; however, it cannot be explained for the full SMA phenotype, implicating not only dysregulated neural networks but other non-neuronal cell types involved in the SMA pathology [10,11]. Emerging research extends the pathogenic effect of SMN deficiency beyond the MN, including other cells inside and outside the central nervous system, so that many peripheral organs and non-neural tissues show pathological changes in preclinical SMA models and diseased patients (Figure 1) [12,13,14]. Furthermore, increasing evidence suggests metabolic abnormalities in patients with SMA, such as altered fatty acid metabolism, impaired glucose tolerance, and muscle mitochondria defects [15,16,17]. Recent studies also indicate that many SMA patients are either undernourished, underfed, or overfed [18]. Notably, in some SMA patients, metabolic dysregulations may even present before their first neuromuscular signs [19]. These findings suggest that SMN is essential for the survival of motor neurons and affects certain enzyme production in the metabolism.

Over the last few years, the increased life expectancy of SMA patients has been achieved through the invention of novel therapies and the standardization of clinical care. However, knowledge of the altered metabolism and nutrition in SMA remains limited. The impact of SMN deficiency on metabolic abnormalities has been recently proposed. Before and after the onset of the disease, metabolomics studies in SMA patients can provide valuable information about the direct or indirect effects of SMN deficiency on metabolic abnormalities [13]. The present review will discuss the current knowledge regarding the metabolic involvement in SMA and the role of metabolomics in pursuing potential biomarkers and therapeutic insights for SMA.

## 2. Lipid Metabolic Abnormalities in SMA

Abnormalities of lipid metabolism have been described in different motor neuron diseases, including SMA [36]. As shown in Table 1, dysregulated lipid metabolism is the first and most studied nutritional problem in SMA [37,38]. Compared with healthy controls and non-SMA motor neuron diseases with equally debilitating statuses, the abnormal lipid metabolism found in patients and animal models appears unique to SMA [39,40]. Abnormal levels of fatty acid oxidation metabolites, especially dicarboxylic aciduria and esterified carnitine, were first reported in several studies of patients with severe SMA type [38,41,42]. Subsequently, an increasing number of studies suggest that patients with SMA are likely to have metabolic defects involving fatty acid metabolism. Of note, increased fat mass, even though relatively low caloric consumption has been repeatedly reported in patients with SMA [40,43]. Several serum fatty acids and lipids have been found correlated to the motor function of patients with SMA, suggesting potential biomarker candidates for SMA [44]. It has recently been implicated that defects in fatty acid transport and mitochondrial β-oxidation may also contribute to muscle wasting in patients with a severe SMA phenotype [32]. Nevertheless, the exact mechanism of this lipid metabolism abnormality in SMA is still unclear, but it is suspected to be related to the absence of the SMN gene product, defects in neighboring genes, or the loss of a neural “trophic factor” [31,42,45].

Although abnormal levels of fatty acid metabolites have been reported, no direct evidence has substantiated a specific defect of mitochondrial β-oxidation in SMA patients. There are several differences in metabolomics between patients with SMA and patients with a genetic defect of fatty acid β-oxidation. SMA patients usually had a normal acylcarnitine profile [42], contrary to an increased acylcarnitine level always found in mitochondrial β-oxidation defects. Moreover, fasting patients with impaired fatty acid β-oxidation always have markedly decreased ketone bodies. However, patients with SMA usually present with a normal or even a high ketone body level (increased ketosis), especially under stress [45,46]. The ability to mount fasting ketosis means that the liver can utilize fatty acids normally, but it does not rule out that it may be caused by muscle-specific mitochondrial defects in β-oxidation [32]. Therefore, it is postulated that dysregulated fatty acid metabolism in SMA patients might be directly related to SMN deficiency but is not attributed to the consequence of major enzyme block of mitochondrial β-oxidation, disuse muscle atrophy, or denervation [13,42,47].

Fatty vacuolization with macro- or micro-vesicular steatosis of the liver has been found in early studies of SMA patients [38,41,42]. Of note, liver failure and Reye-like syndrome with diffuse vesicular steatosis have been recently reported in patients with type 1 or 2 SMA [48,49]. An updated study further reports an increased susceptibility to develop dyslipidemia in 37% of SMA patients, with evidence of liver steatosis in their pathological specimens [27]. Similarly, these human findings are reproduced in different SMA mouse models, of which a specific Smn ^2B/−^ mice model developed the non-alcoholic fatty liver disease (NAFLD) before denervation. Hyperglucagonemia might also contribute to dyslipidemia and hepatic steatosis, possibly through the pancreas–liver axis, leading to peripheral lipolysis of white adipose tissue and an increase in circulating lipids. These findings imply that the liver-intrinsic SMN deficiency might also cause dysregulated metabolism of the hepatocytes [26,50], which could predispose the cells to fat accumulation. Noteworthily, subacute liver failure was recently reported in two patients with type 1 SMA following gene replacement therapy [49]. It is postulated that increased susceptibility to dyslipidemia and associated fatty liver disease could predispose the SMA patient to liver injury, which might be induced or exacerbated after the gene therapy. A thorough investigation of the lipid content in the liver of SMA patients and mouse models, before and after the onset of the disease [47], may provide further evidence for whether the direct or indirect effects of SMN deficiency affect metabolic abnormalities.

Since carnitine and its acyl esters (acylcarnitines) are cofactors for β-oxidation, abnormal lipid metabolism may also be reflected in their production, fractions, and transportation. Because carnitine is essential for intramitochondrial β-oxidation, reduced carnitine would limit β-oxidation. Acylcarnitines are known to play a crucial role in stabilizing neuronal membranes and neurotransmission [51]. Supplementation of acylcarnitine has shown beneficial effects in treating chronic degenerative diseases [52,53]. However, there are still controversies regarding the dysregulation of production, synthesis, and carnitine/acylcarnitine extraction in SMA patients. Early studies suggested that the integrity of nerve and motor neurons might influence carnitine transportation and lipid β-oxidation in muscles. Reduced muscle carnitine and decreased activity of β-oxidation have been observed in animal models after denervation [54,55]. Similarly, reduced carnitine and acylcarnitine levels in plasma and muscles and increased urine excretion of acylcarnitine have been reported in SMA patients [37,56]. However, normal or mild-to-moderate elevated serum acylcarnitines, particularly C5-OH acylcarnitine and C3 propionylcarnitine, were found in the following studies of SMA patients with a severe phenotype [41,42]. In contrast, an updated article reported an adolescent with type 2 SMA who showed a dramatically low serum carnitine/acylcarnitine level at a catabolic state [48]. This finding suggests impaired intramitochondrial β-oxidation associated with dysregulated carnitine metabolism in SMA would become more prominent, especially under stress.

In the fat metabolism of healthy individuals, longer-chain fatty acids are transported into the mitochondria for β-oxidation. Carnitine palmitoyltransferase 1 (CPT1) is an enzyme that converts long-chain acyl-CoA into long-chain acylcarnitine, thereby transporting long-chain fatty acids to the mitochondria. Decreased CPT activity has been reported in muscles of severe type 1 SMA patients, compared with aged-matched infants [56]. Recently, reduced CPT1 activity was also found in an SMA (Smn ^2B/−^) mice model [25]. Of note, an isoform of CPT1, called CPT1c, which mainly expresses in neurons, including motor neurons, shows biosynthetic activity in neuron-specific acyl-CoA. Reduced activity of CPT1c leads to motor function impairment and muscle weakness [57]. Interestingly, an updated study indicates that MN-specific CPT1C can interact with atlastin-1 encoded by the ATL1 gene, which is mutated in hereditary spastic paraplegia, a kind of motor neuron degenerative disorder [58].

Acylcarnitines can also interact with different proteins to influence signaling pathways of neuronal cells [52]. Growth-associated protein 43 (GAP43), a protein involved in neuronal development, neurotransmission, and neuroplasticity, is modified post-translationally by a long-chain acylcarnitine, possibly through the acylation pathway [59]. Interestingly, a recent study found that motor neurons from SMA mouse models showed reduced GAP43 protein levels in axons and growth cones [60,61]. SMN seems to be responsible for regulating the localization and translation of GAP43 mRNA in these axons, and the restoration of GAP43 mRNA and protein levels rescued the defect of axon growth in SMA mice. Therefore, dysregulated acylcarnitine might also affect SMA phenotypes, possibly through the post-translational regulation of motor neuron-specific protein GAP43. Acylcarnitine plays a role in GAP43-related axon growth/repair pathways and may represent a promising SMA treatment target.

Nevertheless, the inconsistent findings of carnitine/acylcarnitine metabolites in SMA patients argue the pathomechanism of the impaired β-oxidation in SMA. Applying modern techniques for quantitative analysis of carnitine and acylcarnitine of various lengths in different samples (e.g., plasma, urine, and muscle) may help decipher this ambiguity [62,63]. However, similar studies in SMA patients are scarce, and the findings of changed carnitine/acylcarnitine levels in SMA patients with different *SMN2* copies have not been updated. The discovery of plasma and urinary metabolite patterns, specifically reflective of fatty acid catabolism, can help clarify biochemical pathways that link lipid metabolism and provide potential biomarkers monitoring disease progression.

## 3. Glucose Metabolic Abnormalities in SMA

The concern about glucose metabolism abnormalities was initially raised through clinical observations in mild-to-intermediate phenotypes of SMA patients (Table 2). Two studies of type 2 SMA patients suggested they might be more likely to experience hypoglycemia following fasting [64,65]. A recent study in type 1 SMA patients also showed a similar finding of hypoglycemia even after a short-term fasting (>4 h but <6 h) [66]. The presence of hypoglycemia after fasting has been postulated to have an association with reduced gluconeogenesis. Because skeletal muscle is an important source of gluconeogenic substrates during fasting, hypoglycemia must be considered for SMA patients with severe muscle wasting, especially during surgery and fever [65]. Therefore, it is recommended that patients with recurrent hypoglycemia episodes should be provided with regular meals based on carbohydrates and protein, including late-night meals.

In contrast, other studies have reported hyperglycemia during fasting in patients with type 2 and type 3 SMA, some of whom were diagnosed with diabetes and ketoacidosis (Table 2) [17,67]. The metabolic syndrome features of increased fat mass and decreased lean mass have been reported in patients with type 2 and type 3 SMA [40]. A recent study also indicated that, in a good state, obese children with SMA type 2 were at increased risk of insulin resistance and impaired glucose tolerance, with 50% of participants showing urinary ketones [16]. It has been postulated that as the skeletal muscle is a major target of insulin action, muscle wasting (sarcopenia) promotes insulin resistance with increased risk of hyperglycemia [68,69]. Additionally, hyperleptinemia has been observed in patients with SMA types 1 to 3, which implies an indirect link to insulin resistance [70]. Nevertheless, even if glucose and insulin metabolism show an increased risk of insulin resistance, HbA1c levels are usually normal in most SMA patients examined [16,69,70].

Similarly, perturbations of glucose metabolism affecting glucose sensitivity and pancreatic defects have been observed in the SMA mice model [17,39,71]. In particular, the metabolic defects in the SMA Smn ^2B/−^ mice model were characterized by fasting hyperglycemia, glucose intolerance, hypersensitivity to insulin, and hyperglucagonemia [17]. In the same study, analysis of pancreatic tissue from infants with SMA type 1 has recapitulated similar pancreatic development defects. Reduced SMN protein levels may also affect the insulin-like growth factor 1 (IGF-1) pathway in the liver of SMA mouse model [72]. IGF-1 is an anabolic hormone with a molecular structure comparable to insulin, which shows myotrophic effects on muscle tissue. Dysregulation of the IGF-1 signaling pathway has also been reported in biopsies from patients with type 1 SMA [73]. A recent study further indicated that IGF1 status is associated with insulin resistance in young SMA patients with early-onset sarcopenia [69]. However, the authors concluded that the myotrophic effect of IGF-1 might be adversely affected by insulin resistance, so therapeutic interventions for dysregulated glucose metabolism in SMA should target insulin resistance.

Nevertheless, it has been suggested that SMA patients receiving partial SMN restoration therapy may increase the risk of having pancreatic and glucose metabolism defects [71]. Meticulous monitoring of glucose homeostasis in SMA patients is essential to clarify the role of SMN in glucose metabolism and pancreatic function.

## 4. Altered Vitamin Level in SMA

A previous study indicates that the activity of SMN depends on folic acid and vitamin B12, both of which are necessary for protein methylation [74]. SMN binds to certain proteins with arginine- and glycine-rich domains, which are modified to dimethylarginine. The binding of other proteins that interact with SMN can also be greatly enhanced by methylation. Inadequate intake of folic acid and vitamin B12 may lead to protein hypomethylation [75], and subsequently may affect the clinical severity of SMA.

The SMN protein may play an active role in bone remodeling or uptake of vitamin D and calcium [35]; therefore, patients with SMA are often accompanied by osteopenia and may contend with fractures due to minor injuries. Compared with other neuromuscular disorders, reduced bone mineral density seems more significant in patients with SMA, especially in those losing ambulatory function [76]. Suboptimal vitamin D intake is frequently observed in patients with all SMA types [18,77]. Low serum levels of vitamin D and 25-OH vitamin D have been reported in patients with type 2 or 3 SMA [34]. However, in a small group of patients with type 1 SMA, the corresponding serum vitamin D levels did not reflect insufficient consumption [78]. Low bone mineral density (BMD) and femur fractures are highly prevalent in all SMA subtypes from a young age; however, few patients met osteoporosis criteria [79]. Adequate bone health assessment and intervention may be an unmet medical need for patients with SMA. It is imperative to determine the natural trajectory of BMD changes at different skeletal sites, especially in adolescent and young adult patients with SMA, and determine if low BMD and propensity to fracture are related to immobility and muscle weakness or direct action of SMN on bone turnover. More work is required to identify effective interventions to delay the decline in BMD and prevent fractures in patients with SMA.

Besides vitamin D and calcium, vitamin E, vitamin K, and folate intakes have been reported below values of Recommended Dietary Allowance (RDA) in half the cohort of patients with SMA [77]. Further research is needed to determine the appropriate intake of vitamin D and other macro- and micro-nutrients in this population.

## 5. Dietary Issues in SMA

Patients with SMA are at higher risk of suboptimal nutrition intake, and nearly half of the cohort demonstrated either undernutrition (underweight) or overnutrition (overweight) over time [18,77]. Changes in body composition, especially the loss of lean body mass, can be particularly harmful to SMA patients because it can impair the respiratory strength of already weak muscles [43]. Therefore, nutrition support is considered a core component of multidisciplinary care for SMA patients [15,80].

However, the specific nutritional challenges in this population are not well described, and a particular diet has not been scientifically evaluated. An early study showed that when the mother was fed a lipid-rich diet, the pups of SMA mice could have a longer survival period and improved motor function [39]. These findings suggested that higher fat content may confer protective benefits during motor neuron loss. However, an updated study reported that low-fat diets could nearly double survival in Smn ^2B/−^ mice, independent of changes in SMN levels, liver steatosis, or enhanced hepatic functions [81]. Although both studies are in the preclinical phase, such controversies suggest a need to establish clinical nutrition guidance from evidence-based research to provide better care for SMA patients.

The advances in therapy for SMA have improved survival and quality of life, which poses new challenges. The survival of patients with severe SMA has generated new phenotypes, and long-term outcomes are unknown [82]. Noteworthily, nutritional management may have a significant impact on the clinical course and even prognosis. For example, previous studies indicated that nutritional support could affect the therapeutic effects of trial agents on different SMA mice models [83,84]. Although it is difficult to show a clear association between metabolic effects in SMA patients who received therapies at this time, it has been emphasized that nutritional care must also be revised and monitored according to individual needs, especially in the SMA therapeutic era [15]. Optimal nutritional management for patients with SMA includes longitudinal evaluation of weight and length and dietary analysis. Recent studies have demonstrated that a modified diet based on measured energy expenditure and optimized protein can improve ventilation and lean body mass in patients with SMA [18,85]. In the future, non-invasive approaches for body composition assessment, e.g., bioelectrical impedance analysis, can be used to evaluate the nutritional status of children with SMA. Further research is needed to assess the use of elemental and semi-elemental formulas in SMA management, including the optimal intake of macronutrients and micronutrients for nutritional support and the ideal fat content and composition.

## 6. Conclusions

Active nutrition support and metabolism surveillance are crucial for patients with SMA, and a comprehensive nutritional assessment could guide individualized nutrition therapy for this vulnerable population. With the emergence of new gene-targeted and disease-modifying therapies, which may affect the metabolism of SMA patients, personalized nutritional optimization may become particularly important. Metabolomics study in SMA patients, before and after the disease onset, may provide valuable information regarding the direct or indirect effect of SMN deficiency on metabolic abnormalities. Furthermore, identifying and quantifying the specific metabolites in biofluids of SMA patients may serve as an authentic biomarker or therapeutic target for SMA.

## Figures and Tables

**Table 1:** Altered Lipid Metabolism in Patients with Spinal Muscular Atrophy.

Altered Metabolic Aspect	Reference	Study Design	Study Aim	Enrollment	Patient Features	Main Findings
Lipid (fatty acid), carnitine	Kelley et al. (1986) [38]	Case report	To describe an SMA infant with elevated certain urinary organic acids, suggesting a defect of fatty acid metabolism.	SMA: 1	SMA type 1; age 9 months old	Increased urine dicarboxylic acids in both fed and fasting states, especially in the longer-chain (C10 and C22) 3-hydroxydicarboxylic acids. Serum carnitine concentration slightly decreased (24 µM/L, free; 37, total). Mild to moderate macrovesicular fatty vacuolization was found in the postmortem liver. Findings could be events not specific to SMA.
Lipid (fatty acid), carnitine, acylcarnitine	Harpey et al. (1990) [37]	Cross-sectional	assess the metabolic defects of fatty acids and carnitine/acylcarnitine among patients with SMA	SMA: 14	SMA type 2: 100%; age range 1–11.5 years old	Urine organic acids showed an abnormal excretion of ethylmalonic acid in all 14 children. Reduced carnitine level in serum of 10 patients and muscles of 6 patients. Increased excretion of urinary acylcarnitines in all examined patients (*n* = 8). Possible multiple Flavin adenine dinucleotide (FAD)-linked acyl-CoA dehydrogenase deficiencies. Carnitine deficiency may be due to intramitochondrial accumulation of acylcarnitines, followed by renal excretion. Findings could be events not specific to SMA.
Lipid (fatty acid), carnitine, acylcarnitine	Tein et al. (1995) [41]	Cross-sectional	To identify and quantify the FA oxidation abnormalities in SMA and to correlate these with disease severity and to identify specific underlying defects.	SMA: 15	SMA type 1: 20%, type 2: 53%, type 3: 27%; age range 2 months old–20 years old	Serum carnitine total/free ratios tend toward an increased esterified fraction ranging 35–58% of total carnitine in children with SMA type 1 and 2. SMA Patients > 23 months old showed normal esterified carnitine levels. Urinary organic acid analysis: abnormalities in SMA type 1 and 2. Mostly normal in SMA type 3. Impaired β-oxidation noted in 5 children (two type 1, two type 2, and one type 3) with a significant reduction in the activities of short-chain L-3-hydroxyacyl-CoA dehydrogenase, long-chain L-3-hydroxyacyl-CoA dehydrogenase, acetoacetyl-CoA thiolase, and 3-ketoacyl-CoA thiolase. All cases had normal crotonase activity. Marked increase in crotonase activity ratios to L-3-hydroxyacyl-CoA dehydrogenase and thiolase activities with short- and long-chain substrates. Findings could be events specific to SMA.
Lipid (fatty acid), carnitine	Crawford et al. (1999) [42]	Plasma studies: cross- sectional Urine studies: two or more single-arm study	To evaluate fasting and non-fasting lipid profiles in urine and plasma in infants and children with SMA.	SMA: 50 healthy controls: 22 disease controls: 6 SMA: 13 healthy control: 23	SMA type 1: 66%, type 2/3: 34% Disease controls: non-SMA denervation disorders (*n* = 6) Healthy controls: age 8–11 months old (*n* = 4), age 1–6 years old (*n* = 19)	Plasma concentration of dodecanoic acid increased in severe SMA. Normal plasma acylcarnitine profiles in 10 infants with severe SMA. The ratio of molar quantities of dodecanoic to tetradecanoic acid differed significantly between severe SMA, age-matched controls, disease controls, and milder SMA type. All severe SMA patients evaluated in the fasting state developed marked dicarboxylic aciduria, including saturated, unsaturated, and 3-hydroxy forms, comparable in magnitude with that of children with primary fatty acid defects β-oxidation. Findings could be events specific to SMA.
Lipid (fatty acid), carnitine, acylcarnitine, ketone, glucose	Zolkipli et al. (2012) [48]	Case report	Describing a type 2 SMA children with catabolic crisis related to possibly impaired intramitochondrial β-oxidation.	SMA: 1	SMA type 2; age 15 years old	Catabolic crisis onset 4 days after surgery, associated with hypoketotic hypoglycemia, lactic acidemia, hyperammonemia and liver failure. No ketonuria. Urine organic acids revealed moderate lactic acid. Low plasma free and total carnitine with a raised esterified fraction. Increase in C6 and C140H serum acylcarnitines after liver transplantation. Liver pathology showed diffuse macro- and micro-vesicular steatosis. The crisis responded in part to mitochondrial therapy and anabolic rescue. Findings could be events specific to SMA.
Lipid (fatty acid), ketone, glucose	Mulroy et al. (2016) [45]	Case report	Describing a type 2 SMA adult presented with severe ketoacidosis with mild hypoglycemia	SMA: 1	SMA type 2; age: 50 years old; BMI: 16.4 kg/m^2^	Reductions in muscle mass, physical stress, and defects in fatty acid metabolism may cause hypoglycemia and non-diabetic ketoacidosis. Findings could be events not specific to SMA.
Lipid (fatty acid), ketone	Lakkis et al. (2018) [46]	Case report	Describing a type 3 SMA adult presented with severe ketoacidosis with normal serum glucose	SMA: 1	SMA type 3; age: 36 years old; BMI: 23 kg/m^2^	Presented with ketoacidosis related to moderate fasting. Denervation or SMN deficiency may affect metabolism and response to hormones, resulting in decreased uptake and fatty acid utilization by muscles. The influence of denervation on muscle β-oxidation may elevate acetyl-CoA, a ketone precursor, in the liver. Findings could be events not specific to SMA.
Lipid (fatty acid), glucose	Deguise et al. (2019) [27]	Cross-sectional	To investigate the lipid profile, including total cholesterol (TC), low-density lipoprotein (LDL), high-density lipoprotein (HDL), non-HDL, and triglycerides to assess abnormalities in fatty acid metabolism To check glucose dysregulation by HbA1C	SMA: 72	SMA type 1 20%, type 2 72%, type 3 8%; median age 3.8 years old	37.5% of SMA patients, most commonly type 1 and 2, had dyslipidemia HbA1C trended lower in most SMA patients, with 57% having an abnormally low readout (HbA1C < 5%) Dyslipidemia and low glucose levels align well with clinical findings in enrolled SMA patients. Findings could be events specific to SMA.

SMA: spinal muscular atrophy.

**Table 2:** Altered Glucose Metabolism in Patients with Spinal Muscular Atrophy.

Altered Metabolic Aspect	Reference	Study Design	Study Aim	Enrollment	Patient Features	Main Findings
Glucose, ketone	Bruce et al. (1995) [64]	Case study	To describe a phenomenon of hypoglycemia in patients with SMA.	SMA: 2	SMA type 2 100%; age 14 years old and 20 years old, respectively	Repeated episodes of hypoglycemia in both cases. One associated with acidosis and ketonuria. Hypoglycemia was explained by reducing muscle protein, leading to lower availability of amino acids (alanine) as substrates for gluconeogenesis in the liver. Regular meals and a late evening meal were recommended in SMA patients with recurrent hypoglycemia. Findings could be events not specific to SMA.
Glucose	Orngreen et al. (2003) [65]	Two or more single-arm study	To investigate the effect of 23 h of fasting on plasma glucose and other metabolites, glucose turnover, and hormonal changes in NMD patients with low muscle mass.	SMA: 4 Healthy controls: 6	SMA type 2 100%; mean age 25 years old; average body weight 29.8 kg Controls: mean age 24 years old; average body weight 69.5 kg	All SMA patients developed hypoglycemia with continued fasting compared to nil hypoglycemia in healthy controls. Patients experienced frequent attacks of headache, nausea, and dizziness that could be alleviated by food intake. Findings could be events specific to SMA.
Glucose, insulin	Bowerman et al. (2012) [17]	Cross-sectional	To describe glucose metabolism and pancreatic developmental defects in SMA.	SMA: 6	SMA type 1 100%; age range 7–35 months old. Control: age range 4–36 months old.	Pancreatic islets from SMA type 1 patients had significantly more alpha cells and fewer beta cells vs. control islets. Islets appeared disorganized in appearance. Pancreatic islet compositions were similar among all patients independent of age. Findings could be events specific to SMA.
Glucose, ketone	Lamarca et al. (2013) [67]	Case study	To describe a phenomenon of DM and diabetic ketoacidosis in a patient with type 2 SMA.	SMA: 1	SMA typ 2, age 29 years old, BMI: 10.2 kg/m^2^	New onset DM presenting with blood sugar: 591 mg/dL), ketonuria (4+) and acidosis (anion gap: 31) Positive family history of type 2 DM (3 patients onset at 60–70 years old) Autoimmune markers for type 1 DM were negative Findings could be events not specific to SMA.
Glucose, insulin, ketone	Davies et al. (2015) [16]	Case series	To examine the impact of fasting and glucose tolerance in an SMA type 2 population.	SMA: 6	SMA type 2 100%; mean age 8.9 ± 1.7 years old (range 7–10 years old)	Five of the six patients demonstrated normal HbA1c, and IGF-1 and one participant had a slightly elevated HbA1c, considered prediabetic. During a 20 h fast, no participant experienced hypoglycemia. At the end of fasting, insulin, alanine, phenylalanine, and branched-chain amino acids were significantly decreased, whereas free fatty acids were increased considerably, and urine ketones were detected in 50% of participants. During an oral glucose tolerance test, 100% of participants showed hyperinsulinemia, and 50% showed impaired glucose tolerance, and 83% showed insulin resistance. Findings could be events specific to SMA.
Glucose	Berti et al. (2020) [66]	Cross-sectional	To describe the incidence of hypoglycemia in type 1 SMA patients after short-term fasting (> 4 h but <6 h)	SMA:45	SMA type 1: 100%; median age: 42 months old (hypoglycemic) vs. 21.5 months old (non-hypoglycemic); BMI: −2.19 kg/m^2^	Hypoglycemia in 17 of 45 patients (5 with fasting for acute illness and 12 with fasting for planned procedure). Main presentations associated with hypoglycemia are hyperhidrosis and tachycardia All symptomatic cases improved after intravenous glucose. Conclude that despite type 1 patients fasting for less than 6 h, hypoglycemia was still common. Findings could be events not specific to SMA.
Glucose, insulin, IGF-1	Brener et al. (2020) [69]	Cross-sectional	To determine the IGF-1 status in SMA patients and its association with insulin resistance.	SMA: 34	SMA type 1: 47%, type 2: 29%, type 3: 24%; mean age: 7.1 years old; mean BMI: −1.60 kg/m^2^	Insulin-resistant patients (*n* = 20) had higher IGF-1 levels compared to insulin-sensitive patients (*n* = 14). Insulin-resistant SMA patients had normal lipid profile and normal glycemic control (HbA1c levels). IGF-1 status is associated with insulin resistance in SMA patients with early-onset sarcopenia. Findings could be events specific to SMA.

SMA: spinal muscular atrophy, DM: diabetes mellitus (DM), IGF-1: insulin-like growth factor-1 (IGF-1).
